# Influence of Sensory Needs on Sleep and Neurodevelopmental Care in At-Risk Neonates

**DOI:** 10.3390/children12060781

**Published:** 2025-06-16

**Authors:** Axel Hübler

**Affiliations:** Hospital for Children and Adolescents, Klinikum Chemnitz, 09116 Chemnitz, Germany; a.huebler@skc.de; Tel.: +49-371-33324100

**Keywords:** neonate, sleep, prematurity, sensory stimuli, early SIDS, kinesthetic, neurodevelopmental care

## Abstract

Objective: The development of a normal sleep–wake rhythm in the first weeks of life depends on the physiological sensory needs of the newborn as well as the environment surrounding them. This includes, for example, avoiding pain, exposure to bright light at night and high noise levels. In high-risk newborns, this process can be influenced by immaturity of the central and peripheral nervous systems, therapeutic strategies and the work organization of an intensive care unit. Methods: This study used a narrative review to examine the literature on the interrelationship of sensory modalities on sleep–wake behavior in the context of neonatal intensive care. The current Cochrane reviews on cycled lighting’s effect on premature infants’ circadian rhythm development and noise or sound management in the neonatal intensive care unit, as well as the World Health Organization (WHO) global position paper on kangaroo mother care, were included. Results: An extensive body of literature relates to fetal and neonatal development of the five sensory modalities: touch, taste, smell, hearing and sight. In contrast, there is a lack of evidence regarding the choice of optimal lighting and suitable measures for noise reduction. Since 2023, the WHO has recommended that, from the moment of birth, every “small and sick” newborn should remain in skin-to-skin contact (SSC) with their mother. Developmental support pursues a multimodal approach with the goal of fostering early parent–child bonding, including the child’s needs and environmental conditions. Discussion: The implementation of early SSC and attention to the sleep–wake cycle require systemic changes in both the obstetric and neonatal settings to ensure seamless perinatal management and subsequent neonatal intensive care. Since there is a lack of evidence on the optimal sensory environment, well-designed, well-conducted and fully reported randomized controlled trials are needed that analyze short-term effects and long-term neurodevelopmental outcomes.

## 1. Introduction

For the newborn, birth leads to a fundamental change in its environmental conditions. An undisturbed adaptation to extrauterine life also requires attention to be paid to developing sensory needs. An environment that allows physiological stimulation of the newborn is a requirement for the development of normal sleep–wake regulation in the first weeks of life. Myers pointed out that sensory processing differences may be an important indicator for those who are more vulnerable to changes in sleep duration when experiencing changes to their surrounding systems and environments [1]. For sick newborns, the neonatal intensive care unit (NICU) may present conditions that interfere with sensory development. These include, for example, bright light exposure [2,3], high noise levels [4,5], potentially painful interventions [6,7,8], intermittent parental presence [9,10,11] and artificial feeding modalities [12,13,14]. In addition to the newborn’s medical conditions, this environment can have a negative impact on stabilization and the developmental prognosis.

The organization of neonatal sleep differs immensely from adult sleep. As described by Wielek et al., its quick maturation and fundamental changes correspond to the rapid cortical development at this age. The neonatal sleep–wake state organization impacts on later development, and sleep characteristics during the first postnatal days are related to cognitive development at the age of 6 months [15,16]. The influence of sleep–wake rhythm on neurological development has also been investigated in later childhood. In a study of preterm toddlers at 1.5 years of age, Ando et al. found that daily variation in wake time, a sleep-regulatory variable, is significantly associated with cognitive development [17]. In children aged between 7 and 11 years, the type of chronotype (morning-type and evening-type) influenced performance, and evening children were expected to be more attentive in the afternoon [18]. When dividing school children into three groups—morning-, intermediate- and evening-type—Figueiredo and Vieira found a greater amount of motor agitation, impulsivity and a higher rate of oppositional behavior in the morning type [18]. For mothers, sleep rhythm and chronotype become increasingly earlier in the first two years after birth [19]. It is unclear whether this is the result of adaptation to their children’s sleep habits or an endogenous process.

The holistic treatment of a high-risk newborn should, therefore, include both the treatment of the underlying disease and the elimination of harmful environmental influences. The lack of sensory stimuli also represents a potential risk, as it is a non-neurotypical situation. A low-stimulation environment in the neonatal intensive care unit (NICU) can lead to alterations in the brain structure and function in hospitalized infants [20]. The Supporting and Enhancing NICU Sensory Experiences (SENSE) program was designed to promote daily, positive and evidence-based sensory experiences for premature infants who spend their first several months hospitalized in the NICU. SENSE is based on a review of published literature from the previous 20 years to identify appropriate, evidence-based multisensory interventions for preterm infants in the NICU. It also aims to encourage parents to provide positive sensory experiences to their infants. [21]. Neurodevelopmental care programs like SENSE can be seen as reducing the factors that disrupt the development of sensory qualities, the central nervous system and circadian sleep–wake regulation.

## 2. Methods

This study used a narrative review to examine the literature on the interrelationship between sensory modalities and sleep–wake behavior in the context of neonatal intensive care. This review’s scope was focused on NICU-based care in premature infants. In addition, studies from the fetal period and full-term infants were used to describe physiological development. Information from animal experiments has been marked accordingly. The current Cochrane reviews on cycled lighting’s effect on premature infants’ circadian rhythm development [2] and noise or sound management in the neonatal intensive care unit [22], as well as the WHO global position paper on kangaroo mother care [23], were included. The websites of the professional societies *American Academy of Pediatrics, European Academy of Paediatrics, German Society of Pediatrics and Adolescent Medicine* and *National Institute for Health and Care Excellence* were searched for recommendations on neurodevelopmental care during neonatal intensive care, as of 29 May 2025.

## 3. Sensory Development and Sensory Environment

**Figure 1:**
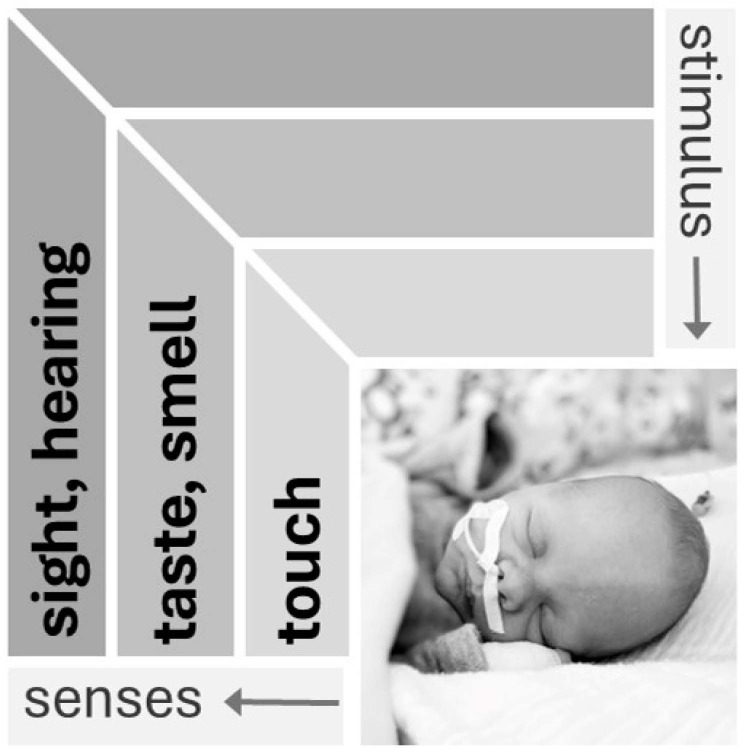
Sensory modalities in the neonate [33]. Senses develop in a species-independent sequence. Sensory properties that determine the immediate interactions of the organism with its environment develop first (touch), and those for orientation in the wider environment develop last (visual) (used with the kind permission of Georg Thieme Verlag).

The sensory development of the five modalities of touch, taste, smell, hearing and sight (Figure 1) influences a newborn’s wakefulness and arousability. Animal models show that sensory development is organized and progressive, with individual elements exhibiting a rapid, invariant and species-independent developmental sequence. This sequence includes (1) skin and joints (touch, pressure, temperature, pain, movement); (2) balance; (3) chemical (smell and taste); (4) hearing and (5) vision. Different species are exposed to this process at different stages of life [24,25].

Based on neurobiological models, the regulation of emotions and behavior can be assigned to three central systems: the brainstem, limbic system and cortex. Circadian rhythm, as a cyclical process, is located in the brainstem together with vagal regulation [26]. The cortical activity typical of arousal and wakefulness is promoted by neurochemical systems that directly innervate the cortex, including the serotonergic regions of the raphe nuclei, dopaminergic neurons of the ventral tegmental area and noradrenergic neurons of the locus coeruleus [27]. The cholinergic neurons of the pedunculopontine tegmental nucleus are active during REM sleep [28]. In neonatal animals, the activity of choline acetyltransferase in the laterodorsal tegmental nucleus is weak in the first week after birth and then increases strikingly in the second week and moderately thereafter [29]. An absence of serotonin in the central nervous system of newborn animals leads to unstable breathing and arousal deficits [30,31]. Sleep state influences how the vagal pathways of the nucleus ambiguus regulate instantaneous changes in heart period in full-term infants [32]. Disturbances of the vagal regulation may therefore substantially affect not only sleep regulation but also the integration of higher-level brain functions.

Before birth, the fetus reacts to touch, smell and sound. Moreover, sonographically, it exhibits facial changes in response to external stimuli. In addition, the fetus is predominantly asleep, partly due to endogenous sedation [34]. Compared to the intrauterine conditions, the extrauterine sensory environment presents fundamental changes to the newborn’s brain. This can lead to malfunctions and disturbances in brain development in premature infants, resulting in neurobehavioral dysfunctions [24]. Differences in the sensory environment of the fetus and neonate are shown in Table 1.

The anatomical basis of **pain perception** develops in the embryo as early as the 6th week of gestation. The synaptic connections for ascending sensory neurons reach the thalamus around the 24th week of pregnancy. The descending inhibitory systems are not fully functional even at birth. Thus, there is still an imbalance between functional ascending excitatory and not fully functional descending inhibitory neurons [33]. The lack of inhibition of superior centers can increase nociceptive transmission in the spinal cord [35] and the perception of pain. Non-painful sensory stimuli can elicit equal or greater physiological stress activation than pain stimuli in preterm and full-term infants. Marchal et al. analyzed behavioral responses to spontaneously occurring sound peaks (SPs) and light level variations (LLVs) through video recordings using the “Douleur Aigue du Nouveau-né” (DAN) scale. SPs of 5 to 15 dBA and LLVs significantly increased the maximum DAN scores compared to baseline. The authors discussed that no definition exists allowing for a distinction between “pain” and “stress” in neonatology [36]. It was not possible for them to exclude that these stimuli induce pain as most of the pain messages are transmitted by non-noxious A-fibers during early stages of development [37]. Repeated pain stimuli may lead to the sensitization of newborns to pain [38] and have an impact on vegetative regulation and pain memory [39,40,41,42]. Chronic or severe pain may affect the functioning of the immune system, potentially making the infant more susceptible to infections and disrupting sleep patterns, leading to poor sleep quality. Crucial for growth, adequate sleep is vital for premature infants [43].

**Table 1:** Differences in stimulus exposure of premature infants compared to the fetal environment (modified according to [44]) *.

Sensory Modality	Intrauterine (Fetus)	Extrauterine (Premature Infant)
**Touch**	Amnion as a protective shell	Direct contact of the immature stratum corneum of the epidermis with the environment
**Taste**	Stimuli are offered via amniotic fluid	Stimuli via teats, disinfectants, care products, different caregivers, food, etc.
**Smell**	Perception of volatile taste components via the amniotic fluid (gustatory smell)	Perception of foreign odors in the air (disinfectants, olfactory signature of the practitioner, food, incubator…); possible toxicity of individual substances
**Hearing**	Perception of predominantly low-frequency sound waves via bone conduction; own mother’s voice is continuously present, external and body sounds of the mother mix	Perception of all frequency ranges via air conduction; background noise includes technical equipment of intensive care medicine and conversations of different people; voice of own mother inconsistently present
**Sight**	Optical input extremely limited; retina and lens developing; functional immaturity of the visual cortex; influence of the mother’s circadian rhythm	Visual environment varies from almost complete darkness to strong brightness; in intensive care, there is little day–night difference; retinal pathology due to high oxygen partial pressure

* Used with the kind permission of Georg Thieme Verlag.

The **acoustic environment** of the traditional neonatal intensive care unit (NICU) is significantly different from the intrauterine environment in the last third of pregnancy. All frequency ranges are available because sounds are transmitted through the air. Noise can affect the neonate for hours throughout the day without circadian rhythms or changes in intensity [4]. The ability to discriminate an acoustic signal from background noise develops later in childhood. The effects of interventions, such as playing the mother’s voice through playback devices [45], on long-term physical or mental development are not sufficiently proven.

The development of **vision** in the fetus is limited by retinal and lens maturation. Only a small amount of light reaches the eye prenatally. Studies on primates suggest that this small amount of light may be sufficient to cause changes in brightness throughout the day [46]. Following the example of its own mother, the fetus develops a circadian rhythm and a number of biological functions (such as growth hormone secretion, heart rate and temperature control, renal calcium excretion and noradrenaline synthesis) [47]. Shortly before the actual birth date, a conserved intrinsic program leads to a switch in cortical visual processing from a so-called “bursting period” to continuous cortical activity [48]. Vision undergoes its most rapid development after birth. Before the 28th week of pregnancy, external visual stimulation is very unlikely to be of any benefit to the infant, but it could interfere with the development of other senses [49]. In animal studies, complete light deprivation during the neonatal period leads to structural and functional changes in the visual system [50].

## 4. Postnatal Sleep Characteristics

### 4.1. Development of Sleep–Dream Cycle and Sleep Stages in the First Weeks

The newborn shows a polyphasic sleep pattern and spends about 18 to 20 h of the day sleeping. A circadian rhythm becomes apparent around three months of age. By one year, an infant already spends most of the day awake. The typical electroencephalographic (EEG) features of the two different sleep stages, rapid eye movement (REM) and non-REM sleep, which are found in older children and adults but are not detectable in newborns.

The sleep stages of newborns can be differentiated into the following categories: quiet sleep, active sleep, transitional sleep and awake [51]. If the sleep stage cannot be classified, it is called indeterminate [52]. In awake full-term infants, continuous baseline activity can be detected in the EEG. During quiet sleep, delta activity occurs, which is referred to as the slow-wave sleep pattern (SWS). Discontinuous baseline activity (tracé discontinue) is characteristic of the EEG in very premature infants [53,54]. The maturation of REM sleep in early infancy may be associated with the presence of melatonin. Prior to the age of 3 months, plasma melatonin levels are low and the characteristic circadian rhythms of melatonin are absent [55].

Electrophysiological examinations cannot replace clinical assessment. For example, the concordance between EEG parameters and observed sleep stages depends on the maturity of the newborn [56]. Clinical observation should be an essential part of any assessment of a newborn’s vigilance. More than fifty years ago, Prechtl described six behavioral states of the newborn, including quiet and active sleep [57,58]. Failure to consider these behavioral states can lead to errors in neuro-neonatal examination [59]. For instance, startles and chin movements are normal phenomena in quiet sleep, but are suspicious for seizures in other stages. Twitching of the extremities, eye movements and reduced muscle tone are features of active sleep. Before the introduction of video recordings, these behavioral states were documented during polysomnography by a pediatric nurse who observed the infant.

Full-term newborns spend about half of their sleep time in active sleep. The percentage of sleep time spent in REM sleep is far greater in preterm than in term neonates. Extremely preterm neonates not only sleep around 97% of the day, but also spend 80% of this time in active sleep [51]. The maturation of the sleep–wake rhythm occurs faster in premature than in full-term infants [60]. Nevertheless, premature infants show more frequent sleep problems after six months of life than full-term infants and polysomnographic measurements continue to show an increased incidence of apneas [61].

Roffwarg et al. stated in 1966 that “*The prime role of ‘dreaming sleep’ in early life may be in the development of the central nervous system*” [62]. A reduced time spent in REM sleep during early infancy has been shown to have lasting effects on later neurocognitive functioning. Studies have often focused on active sleep and its role in early brain development, but quiet sleep and the quality of the sleep–wake cycle seem to be essential for preserving brain plasticity [63].

Gamma-aminobutyric acid (GABA) is the main inhibitory neurotransmitter in the central nervous system. In adult humans (and rats), GABA_A_ receptors are ligand-activated chloride channels, and the influx of chloride into neurons causes hyperpolarization. In early developmental stages, GABA receptors lead to an efflux of chloride, the depolarization of neurons and excitation. The exact time when GABA switches from having an excitatory to an inhibitory quality is not known [64,65]. De Groot et al. recently suggested that the brain seems to be more attuned to endogenous stimulation when GABA has a depolarizing effect, while it is more attuned to exogenous stimulation when GABA has a hyperpolarizing effect. If the brain has not yet undergone the GABA shift, which may be the case in premature infants, an increase in exogenous sensory stimulation can disrupt ongoing neurological developmental processes [66].

### 4.2. Sleep Monitoring in the Neonate

The analysis of sleep depends on the measurement of multimodal parameters. In clinical routine, this requires the installation of additional electrodes or adhesive patches and cables. Software solutions already allow the integrated evaluation of various parameters collected using variable measurement systems such as cardiovascular monitors, video surveillance, EEG or amplitude-integrated EEG (aEEG) [67]. There are interesting approaches in the research for recording vegetative parameters using contactless devices such as radars [68].

**Bedside cardiovascular monitoring** is applied continuously in the neonatal intensive care unit. The parameters recorded during oxycardiorespirography (OCRG) enable the diagnosis of hypoxemia, apnea and bradycardia at the bedside. The internal memory of a monitor and trend analyses allow for a preliminary quantification of events, the detection of periodic breathing and the classification of disturbances in relation to daytime [54,69]. Documentation of events is also of great importance. In everyday clinical practice, approximately only 23% of desaturations and 60% of bradycardias are currently documented by medical staff using bedside monitoring [70].

**The normal values for cardiovascular parameters** depend on the gestational age of the infant. Heart rate and respiratory frequency are strongly correlated with active sleep and quiet sleep. In quiet sleep, as compared to active sleep, respiratory frequency is more stable and the heart rate is lower and less variable [71]. Bohnorst et al., after measuring full-term infants in quiet sleep, reported that desaturations, apneas and bradycardias below 80 beats per minute were common [72]. In healthy-term neonates born at 37 to 41 weeks of gestation, the median SpO_2_ was 95.4% [73]. Premature infants had twice the incidence of desaturations compared to full-term infants, and these desaturations were deeper. The incidence of longer desaturations correlated with healthcare utilization over the first 24 months [74]. Reference values are given in Table 2.

The optimal target range of pulse oximeter oxygen saturation for preterm infants remains controversial. The currently used limits are higher than the former limits, while the most extreme limits have almost been abandoned [76]. A neonatal oxygenation prospective meta-analysis of oxygen target ranges (85–89% versus 91–95%) showed that assignment to the higher target range increased the risk of retinopathy but reduced the risks of death, severe necrotizing enterocolitis [77] and bronchopulmonary dysplasia [78].

There is no consistent definition for the duration of apnea of prematurity (AOP). The information ranges from “... rather arbitrarily set at 4 s ...” [79] to “apnea with heart rate decreases to <100 beats per minute, or drop in SpO_2_ to <80%, or apnea length of >20 s” [80]. Apnea becomes clinically significant when it is accompanied by bradycardia and/or hypoxemia.

Continuous monitoring of transcutaneous CO_2_ (tc CO_2_) levels can provide additional information on respiratory stability, especially in newborns receiving invasive or non-invasive respiratory support in the NICU as well as during polysomnography. The median range of tcCO_2_ in ventilated neonates is 50.4 + 20.4 mmHg [81].

**Polysomnography** (PSG) is considered the diagnostic “gold standard” in pediatric sleep medicine, and can also be performed on premature infants. The practical application of PSG involves the synchronous recording of a variety of parameters. These include electrocardiograms, pulse oximetry and multiple EEG channels (often three on each side, not the complete ten–twenty system). The evaluation is still carried out manually, mostly in 30 s sections, referred to as epochs. In addition to the EEG, other parameters—such as breathing, heart rate and video monitoring—are used for sleep stage analysis.

### 4.3. Early SIDS

The actual definition of **SIDS** (sudden infant death syndrome) remains unchanged from Beckwith’s old description: “The sudden death of any infant or young child which occurs unexpectedly and in which a careful postmortem examination does not reveal an adequate cause of death.” [82]. Similar descriptions can be found in the literature that describe the condition in a similar way: “sudden unexpected death in infancy” (SUDI) [83], “sudden infant death” (SID) [84]. “A brief life-threatening but resolved event in an infant is called “sudden apparent life threatening event” (**S-ALTE**) [85] or “brief resolved unexplained event” (BRUE) [86].

The incidence of S-ALTE on the first day of life is reported to be 2.6 per 100,000 live births. Newborns can be affected immediately after birth with the following risk factors: mother’s first delivery (80%), first 2 h after birth (70%), vaginal birth and position on the mother’s chest or stomach (“potentially asphyxiating position“) [87]. In cases of S-ALTE, resuscitation to stabilize vital functions must be initiated according to the current guidelines [88].

Without a death scene investigation, a SIDS diagnosis cannot be confirmed [89]. Postmortem examinations may show missing pathological findings or varying degrees of organic changes. Diagnosis is now possible for some previously unexplained deaths. For example, long QT syndrome, with a prevalence of 1 in 2000 live births, probably contributes to approximately 10% of sudden deaths [90].

The current guidelines on the **prevention of sudden infant death syndrome** from the German Society for Sleep Research and Sleep Medicine include various risk reduction elements [91]. The special needs of premature infants during neonatal intensive care partly contradict some of these recommendations. In the first weeks of life, lying in the prone position with the upper body slightly raised improves respiratory stability, although “sleeping the infant in the supine position on a firm surface” is recommended. Early and clear communication between the treatment team and parents is important to ensure the transition to home care and compliance with recommendations. Implementation of active public health programs during the neonatal period [92] and acceptance among the target group [93] play an important role in reducing the risk of SIDS.

**Arousability** is considered to play a role in the pathogenesis of SIDS. An arousal is characterized by an abrupt frequency acceleration of at least 1 Hz in the EEG with a minimum duration of 3 s. It is not an actual awakening reaction, but rather a discreet alarm signal from the organism for its own protection [69]. An increased threshold (reduced arousability) is considered a risk factor for SIDS [94], while an increased number of arousals lead to sleep fragmentation and a disrupted sleep cycle. The final pathway is cortical activation [95]. In premature infants, reduced arousal rates at the actual date of birth are associated with perinatal brain damage [96], whereas at two to three months of age, they are associated with the prone position [97].

## 5. Bonding and Kangaroo Mother Care

Pregnant women develop mental representations of their unborn child. The resulting “materno-fetal bond” intensifies during pregnancy [98]. The expectant mother develops feelings and a sense of belonging to her unborn child. From an anatomical point of view, humans have subcortical neuroendocrine structures which, as in all other mammalian species, are associated with basic behaviors (maternal aggressive instincts) and basic emotional states (joy) [99]. Shortly after birth, there is a sensitive period that appears to have long-term effects on maternal bonding and can ultimately affect the child’s development [100]. With this in mind, is it appropriate for a newborn to be separated from its mother immediately after birth for medical monitoring and treatment?

Researchers in the United States investigated the effects of “extra contact” between mothers and their newborns after birth in the early 1970s [101]. This “extra contact” consisted of the skin-to-skin holding of the neonate on the mother’s bare chest as soon as possible after birth. Rey and Martinez began investigating the same care method in Bogotá, Colombia, in the mid-1970s and called it “kangaroo care” [102] or “kangaroo mother care” (KMC). At that time, it was also shown that, from the point of view of the temperature stability of the newborn after birth, separation from the mother is not necessary [103]. Since the 1990s, the positive effect of KMC and medical safety have been reported [104,105,106,107], as well as evidence for the method reducing in morbidity, mortality and nosocomial infection [108].

In 2023, the World Health Organization (WHO) published a Global Position Paper on KMC that is applicable to all countries: from the moment of birth, every “small and sick” newborn should remain with mother in immediate and continuous skin-to-skin contact (SSC), receiving all required clinical care in that place [23]. This recommendation was primarily based on the results of a randomized controlled trial (RCT) of 3211 newborns and their mothers. Newborns with birth weight between 1.0 and 1.799 kg who received immediate kangaroo treatment had a lower mortality rate after 28 days than those who received conventional treatment [109]. In preterm infants between 28 and 31 weeks of gestation, two hours of skin-to-skin contact between the mother and newborn in the delivery room did not improve neurodevelopmental outcomes at 2 to 3 years of age, but skin-to-skin contact improved breastfeeding practices up to 12 months compared with standard care [110]. KMC can improve cardiorespiratory stability in ventilated preterm infants. In an observational study by Sehgal et al., oxygen desaturations and fluctuations in heart rate and bradycardia were less frequent in KMC compared to incubator care [111].

Skin-to-skin care on the first or second day of life does not increase the risk of intraventricular hemorrhage and sepsis in extremely premature infants [112]. A meta-analysis of nine studies showed a significantly positive effect of KMC on body temperature stability and oxygen saturation [113]. A systematic review of 31 studies with 15,559 newborns showed a lower risk of mortality when KMC rather than conventional care was used. The benefits were greater with early initiation and at least eight hours of daily KMC than with late initiation or shorter-duration KMC [114]. Skin-to-skin care in newborns with an umbilical vein catheter does not cause complications compared remaining in the incubator and can be considered safe [115].

The risk of harm from skin-to-skin care or KMC probably lies not in the method itself but rather in its implementation. For monitoring the newborn under KMC, oxygen saturation is a more reliable method than use of an electrocardiogram because superimposed electric activity from the parent can lead to errors [116]. The most common cause of sudden unexpected postnatal collapse is the poor positioning of the newborn during skin-to-skin contact or breastfeeding when the newborn is not being observed by a health professional, attentive parent or caretaker [117]. Bergman et al. pointed out that the implementation of KMC requires systemic changes in both obstetric and neonatal settings to ensure seamless perinatal care [118].

## 6. Sleep Hygiene and Neurodevelopmental Care

The development of a circadian rhythm is potentially influenced by internal and external factors. Supporting it requires a multimodal approach (Table 3).

The adjustment of **lighting levels** in patient rooms should involve the use of moderate daylight during the day and a dark environment at night. In 2024, Morag et al. questioned whether cycled light (CL) is preferable to near-darkness (ND) or continuous bright light (CBL) in fostering development and maturation and reducing adverse neonatal health outcomes. Their systematic review of 20 studies in 1633 infants showed that, compared to CBL, CL may reduce the duration of initial hospitalization, but the evidence is very uncertain. It is not known whether cycled light makes a difference to a baby’s growth, nervous system development, length of oxygen treatment or unwanted side effects compared to CBL [2].

In the NICU, the operating **noise** of the equipment causes higher noise levels than the recommended maximum of 45 decibels [5]. When using major neurodevelopmental disability as primary outcome, Sibrecht et al. identified only one RCT that enrolled 34 newborn infants randomized to the use of silicone earplugs versus no earplugs for hearing protection. No studies evaluated interventions to reduce sound levels below 45 dB across the whole neonatal unit or in a room within it. As there is a lack of evidence, large, well-designed, well-conducted and fully reported RCTs are needed to analyze different aspects of noise reduction in NICUs [22].

**Human milk** (HM) contains several bioactive components with chrono-biotic characteristics. HM has the potential to function as a “synchronizer”, helping the infant to consolidate a circadian sleep–wake cycle. As Paditz recently described, it is still unclear why the pineal gland is not able to initiate its own pulsatile synthesis and the secretion of melatonin in the first months of life. As a result, during this time infants are dependent on an external supply of melatonin [119]. Melatonin, tryptophan, nucleosides/nucleotides and vitamin B12 are components of HM that have sleep-promoting characteristics [120].

**Medical staff** play a key role in meeting the needs of the newborn. The treatment team can help with simple rules to promote the development of a day–night rhythm in premature infants. These rules mean that planned interventions are carried out during the half of the day from 8 a.m. to 8 p.m. This also includes extubation attempts after long-term ventilation.

**Table 3:** Considerations for improving sleep hygiene in the neonatal intensive care unit.

Condition	Suggestion	Practical Aspects
**Lighting conditions**	Lighting adapted to the time of day	Light transmission to the visual system is possible even when the eyes are closed Use of moderate daylight during the day and a dark environment at night No direct exposure to light (stress reactions, unphysiological activation)
**Background noise**	Avoiding high noise levels	Limitation of communication directly in the patient’s unit [121] Ensure sufficient rest periods during intensive therapy
**Parent–infant bond**	Spend sleep phases as part of kangaroo care	From the moment of birth, every “small and sick” newborn should remain with mother in immediate and continuous skin-to-skin contact [23]
**Work organization**	Internal ward rules for activities on patients	Day half (“from 8 a.m. to 8 p.m.”): planned interventions Night half (“from 8 p.m. to 8 a.m.”): only regular care Extubation attempts after a longer ventilation should be carried out during the day
**Nutrition**	Promoting breastfeeding	Attention to human milk’s potential to function as a “synchronizer” due to its several bioactive components [119]

## 7. Discussion

There is a lack of evidence regarding the adequate sensory stimulation of at-risk neonates in the promotion of physiological sleep behavior. A better understanding of the development of circadian rhythms in extreme prematurity is needed. This includes, for instance, the chronobiological effect of nutrition, the GABA shift and the meaning of arousals. An “ideal” technique for monitoring vital functions during intensive care should be contactless. Analysis of data using artificial intelligence could allow for individualized treatment. The aim of these efforts should be to reduce developmental risks for the newborn and enable early bonding with its mother.

As discussed in a review by Harvey recently, research found inconsistent support for cycled lighting depending on the age at initiation when assessing weight gain, quality of movement, sleep and activity rhythms, ventilator days, length of stay, time to oral feeding and irritability [122]. Before 28 weeks of pregnancy, external visual stimulation is unlikely to be of benefit to the premature infant [49].

Effective noise reduction for premature babies seems only possible in conjunction with technological developments. This particularly applies to the incubator as a resonance cavity. The study of Reuter et al. focused on noise level and sound characteristics within the incubator. They measured high noise levels related to various real-life situations within the NICU environment. A strong tonal booming component was noticeable, caused by the resonance inside the incubator. The authors discussed that sound characteristics, the strong low-frequency incubator resonance and levels in decibel (dB) should be at the forefront of both the development and promotion of incubators when helping to preserve the hearing of premature infants [123].

Smartphone-based applications are beginning to be used in the clinical care of newborns, for example, for monitoring resuscitation [124,125], telemedicine consultation [126], retinopathy screening [127,128] and bilirubin monitoring [129,130]. Digital devices are also used intensively by parents during their stay beside the incubator, as medical staff in every neonatal intensive care unit can probably report. To date, there is no systematic information on how this new behavior affects the parent–child bond, nor on the influence of this additional source of light and noise on the neonate.

The use of chronobiologically adjusted infant formula milk seems to be effective in improving the consolidation of the circadian sleep–wake cycle in bottle-fed infants [131]. In contrast to these considerations, probiotic supplementation does not increase infant sleep duration within the first four postpartum weeks, but may contribute to reduced sleep duration in the fifth week [132].

To date, there is an unresolved contradiction between atypical neurosensory stimulation and sensory deprivation. Atypical neurosensory stimulation can interfere with the development of the other senses. The lack of external sensory stimuli, in turn, can inhibit the development of sensory skills.

Intervention programs in neonatal intensive care units are based on different multidimensional approaches. For instance, the Newborn Individualized Developmental Care and Assessment Program (NIDCAP) developed by Heidelise Als (1940–2022) uses individualized care plans based on developmental assessments of an infant’s behavior [133,134,135]. A systematic review including 627 preterm infants did not find any evidence that NIDCAP improves long-term neurodevelopmental or short-term medical outcomes [136].

The supporting and enhancing neonatal intensive care unit sensory experiences (SENSE) program, based on interventions such as massage, auditory exposure, rocking, holding and skin-to-skin care, can help parents to provide positive sensory experiences. The authors of the concept made it clear that “more research is needed” based on the current lack of evidence [137].

The debate regarding the benefits of these intervention programs continues in the neonatal community. Recently, Ahlqvist-Björkroth et al. stated that they considered NICU parenting interventions to be beneficial for both parents and their infants, even if there are knowledge gaps and the interventions are based on different theoretical backgrounds [138].

## 8. Conclusions

Until now, there has been no randomized controlled trial that addresses the impact of sleep-improving interventions in preterm infants on short-term morbidity and long-term neurodevelopmental outcomes.

There is a lack of evidence regarding the optimal sensory environment for premature infants in terms of noise and light exposure.

The benefits of holistic neurodevelopmental programs, including sleep hygiene, are not proven, and more research is needed.

Until better-designed studies are available, treatment teams must individually consider how to avoid sensory overstimulation or deprivation in relation to sleep hygiene for premature infants.

## Figures and Tables

**Table 2:** Reference values for heart rate, respiratory rate and oxygen saturation in full-term and late-preterm neonates.

	Heart Rate [bpm] ^1^	Respiratory Rate [rpm] ^2^	Oxygen Saturation [%]	Ref.
Full-term ^3^	87–133 (112)	32–57 (44)	94–100 (98)	[72] *
Late-preterm ^4^	102–164	15–67	94–100	[75] **

^1^ bpm = beats per minute; ^2^ rpm = respiratory rate per minute; ^3^ in quiet sleep; ^4^ no sleep stage noted. * 3rd and 97th percentiles (50th percentiles in squares) of cardiorespiratory parameters for full-term neonates. ** reference ranges of heart rate, respiratory rate and oxygen saturation in late-preterm neonates.
